# Biotechnological Potential of *Araucaria angustifolia* Pine Nuts Extract and the Cysteine Protease Inhibitor AaCI-2S

**DOI:** 10.3390/plants9121676

**Published:** 2020-11-30

**Authors:** Roberto Carlos Sallai, Bruno Ramos Salu, Rosemeire Aparecida Silva-Lucca, Flávio Lopes Alves, Thiago Henrique Napoleão, Patrícia Maria Guedes Paiva, Rodrigo da Silva Ferreira, Misako Uemura Sampaio, Maria Luiza Vilela Oliva

**Affiliations:** 1 Departamento de Bioquímica, Universidade Federal de São Paulo, Escola Paulista de Medicina, Rua Três de Maio 100, São Paulo 04044-020, SP, Brazil; roberto.sallai@fsa.br (R.C.S.); brsalu@unifesp.br (B.R.S.); pelopes2@yahoo.com.br (F.L.A.); rodrigobioq@gmail.com (R.d.S.F.); misakosampaio@gmail.com (M.U.S.); 2 Centro Universitário Fundação Santo André, Av. Príncipe de Gales, 821, Santo André 09060-650, SP, Brazil; 3 Centro de Engenharias e Ciências Exatas, Universidade Estadual do Oeste do Paraná, Unioeste, Campus de Toledo, R. da Faculdade 645, Toledo 85903-000, PR, Brazil; roselucca@gmail.com; 4 Departamento de Bioquímica, Universidade Federal de Pernambuco, Av. Moraes Rego, S/N, Cidade Universitária, Recife 50670-420, PE, Brazil; thiago.napoleao@ufpe.br (T.H.N.); patricia.paiva@ufpe.br (P.M.G.P.)

**Keywords:** *Araucaria angustifolia*, bioactive compounds, cysteine protease inhibitor, functional food, insecticide, plant extracts, termites, tumor cells, pine nuts, urban pest

## Abstract

Protease inhibitors are involved in the regulation of endogenous cysteine proteases during seed development and play a defensive role because of their ability to inhibit exogenous proteases such as those present in the digestive tracts of insects. *Araucaria angustifolia* seeds, which can be used in human and animal feed, were investigated for their potential for the development of agricultural biotechnology and in the field of human health. In the pine nuts extract, which blocked the activities of cysteine proteases, it was detected potent insecticidal activity against termites (*Nasutitermes corniger*) belonging to the most abundant termite genus in tropical regions. The cysteine inhibitor (AaCI-2S) was purified by ion-exchange, size exclusion, and reversed-phase chromatography. Its functional and structural stability was confirmed by spectroscopic and circular dichroism studies, and by detection of inhibitory activity at different temperatures and pH values. Besides having activity on cysteine proteases from *C. maculatus* digestive tract, AaCI-2S inhibited papain, bromelain, ficin, and cathepsin L and impaired cell proliferation in gastric and prostate cancer cell lines. These properties qualify *A. angustifolia* seeds as a protein source with value properties of natural insecticide and to contain a protease inhibitor with the potential to be a bioactive molecule on different cancer cells.

## 1. Introduction

*Araucaria angustifolia* is a native gymnosperm of the greatest economic and biological importance in Brazil. It withstood the rigors of the natural selection process for hundreds of millions of years as the planet underwent intense geological and climate change. Because of its wide distribution in Parana state, this species is its state symbol and known for Paraná pine. Uncontrolled logging and the expansion of new agricultural areas harmed the forests harboring these trees to such a critical point that *A. angustifolia* is now on the official list of endangered species of the Brazilian flora and the red list of the International Union for Conservation of Nature [1]. *Araucaria* seeds have a remarkable structure, whose development is controlled directly or indirectly by changes in gene expression patterns and is an interesting biological model for cellular organization studies, protein accumulation, and differential gene expression. In addition to starch (36.28%), proteins (3.57%), lipids (1.26%), carbohydrates (2.43%), and minerals provide high nutritional value [2]. The reddish-brown peel and the thin film are rich in polyphenolic compounds with antioxidant properties that, when transferred to the edible part during the cooking process, make it a very healthy food product. In forests, the pine nuts of Araucária are a key food for various vertebrates such as agouti, squirrels, monkeys, rodents, and various species of birds [3].

The overall seed characteristics and a variety of diverse structures have led researchers to seek new substances with anti-tumor activity and therapeutic effects on various diseases [4,5]. A few studies have been performed with *A. angustifolia*, and lectins having anti-inflammatory, antibacterial, and anti-depressant action on the central nervous system were isolated [6,7].

Plants synthesize numerous proteins that contribute to the protection against attack by microorganisms (fungi and bacteria) and/or invertebrates (insects and nematodes). In most cases, the biological role of these proteins is assigned based on their in vitro activity, as is the case with lectins and enzyme inhibitors. In other cases, their role is confirmed by more direct analysis such as the incorporation of these in artificial diets used in insect feeding, their incorporation into culture media for microorganism culture, or even through the expression of these proteins in transgenic plants [8,9].

Inhibitors of serine proteases have been known when Kunitz and Bowman in 1946, and Birk in 1963 purified and characterized trypsin inhibitors from soybean seeds [10]. Since then, inhibitors have been isolated mainly from reserve organs such as seeds and tubers. Cysteine protease inhibitors or cystatins are reversible protease inhibitors of the papain family and related proteases (e.g., cathepsin B, L, ficin, and bromelain). In plants, orizacystatin from rice seeds was the first inhibitor of cysteine proteases considered a cystatin [11]. Numerous biological functions attributed to phytocystatins have recently been reviewed. It is assumed that they may play a regulatory role in all physiological processes involving cysteine proteases. More systematic studies have led to very promising results, especially the use of such inhibitors as instruments for the study of protease involvement in pathophysiological processes [12,13].

Phytocystatins have been identified in a large variety of monocotyledons such as rice, corn, maize, barley, and sugarcane, and dicotyledons such as beans, potatoes, avocados, kiwis, and nuts [8,14,15]. Fewer reports exist on the purification of cysteine protease inhibitors. In this work, we describe the biotechnological potential of *Araucaria angustifolia* pine nuts on phytopathogenic organisms, extending structural and functional characterization of a cysteine protease inhibitor toxic for human tumor cell lines improving the qualification of the nuts as a functional food.

## 2. Results

To minimize possible proteolysis, the extract was heated at 60 °C for 15 min, and the inhibitory activity of the cysteine proteases papain, cruzain, and human L-cathepsin was preserved. No inhibitory activity was detected on cathepsin B or serine proteases, such as trypsin, human plasma kallikrein, porcine pancreatic elastase, or human neutrophil elastase.

### 2.1. Effect of Extract on Adult Insects

Protease inhibitors have demonstrated insecticidal activity by interfering with digestion, which leads to poor nutrient absorption and decreased amino acid bioavailability [16]. Thus, we investigate the protective effect of pine nuts on adult termites. 

**Figure 1:**
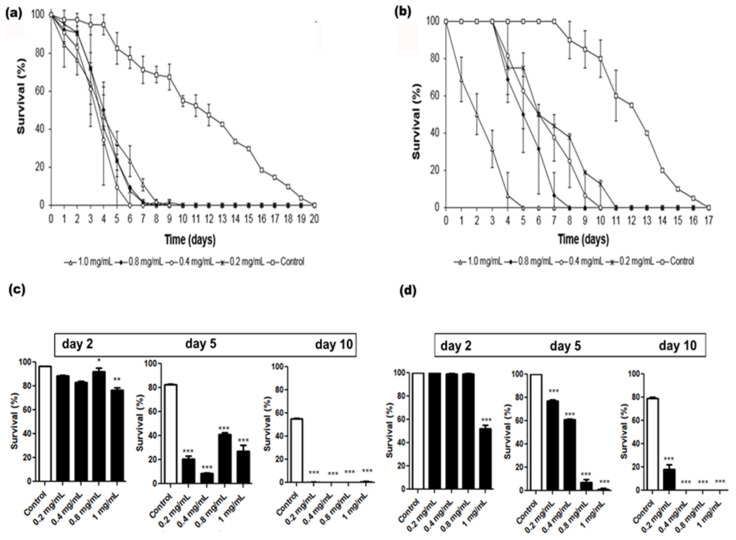
Effect of *A. angustifolia* seed extract on the survival of *Nasutitermes corniger* workers (**a**,**c**) and soldiers (**b**,**d**) during 20 days. Saline solution (0.15 M NaCl) was used in negative control. Each point represents the mean ± standard deviations of three repetitions. (* *p* < 0.05, ** *p* < 0.005, *** *p* < 0.0001; one way-ANOVA, follow Tukey’s multiple comparison test).

The extract exhibited termiticidal activity on *N. corniger* workers at all tested concentrations (Figure 1a). All the workers died after 10 days in the treatments with extract while 100% mortality in negative control was reached only until the twentieth day. No significant differences (*p* > 0.05) were detected between the effects of the concentrations tested. Regarding the effect on soldiers, the extract was also able to kill the insects at all tested concentrations (Figure 1b); in negative controls, 100% mortality occurred only on the seventeenth while in the treatment at 1.0 mg/mL, for example, all insets had died on the fifth day.

### 2.2. Purification of the Cysteine Protease Inhibitor AaCI-2S

**Figure 2:**
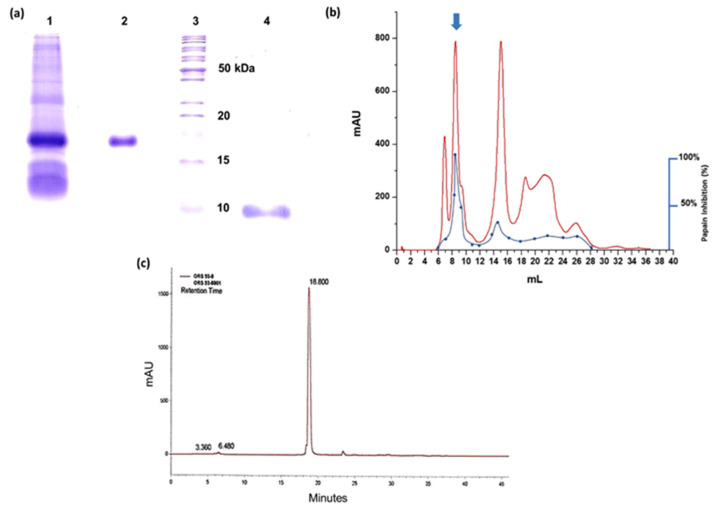
Purification profile of the AaCI-2S inhibitor. (**a**) SDS-polyacrylamide gel electrophoresis (15%). Lane 1, Brazilian pine saline extract (100 µg); Lane 2, non-reduced AaCI-2S (20 µg); Lane 3, molecular mass markers; Lane 4, AaCI-2S (10 µg) under reducing conditions. (**b**) Superdex 30 column equilibrated with 0.05 M Tris-HCl buffer (pH 8.0) containing 0.15 M NaCl at a flow rate of 0.5 mL/min. Absorbance at 280 nm is indicated in red and the inhibitory activity on papain in blue. The arrow indicates the fractions pooled. Sample: protein (2 mg A280) after ion-exchange chromatography. (**c**) Reverse-phase chromatography Vydac C-18 column. The proteins were eluted with an acetonitrile gradient in 0.1% TFA.

The characterization of the cysteine protease inhibitor became the focus of the present study because it is not much studied in gymnosperms, like the inhibitors of serine proteases. The acetone-fractionated proteins from the saline extract (Figure 2a, line 1) were separated by chromatography using a DEAE-Sephadex anion exchange resin followed by the cation exchange chromatography in SP-Sephadex. In the DEAE-Sephadex anion exchange resin, the inhibitor did not bind under the buffer at pH 8. Even with the change in ionic strength and pH parameters, the chromatographic profile was not modified, and most of the inhibitory activity was detected in the non-bonded material eluted with the column equilibration buffer. Using the same buffering conditions, the inhibitor also did not bind to the cationic resin SP-Sephadex. The papain inhibitory activity was detected after the chromatographies were dialyzed, lyophilized, and loaded on a Superdex 30 column in an ÄKTA purifier system (GE Life Sciences, USA). Figure 2b shows the protein profile and the location of the inhibitory activity indicated by the second peak. Fractions with inhibitory activity were pooled and analyzed by SDS-PAGE and reverse-phase chromatography. The estimated molecular mass of the inhibitor was around 18 kDa (Figure 2a, lane 2), and under reducing conditions, it showed a unique band of approximately 9 kDa (Figure 2a, lane 4). Reverse-phase chromatography onto a C-18 column in an HPLC system (Figure 2c) exhibited the presence of a single major peak, indicating the purity of the preparation.

### 2.3. Secondary Structure Estimation and Intrinsic Fluorescence Emission of AaCI-2S

**Figure 3:**
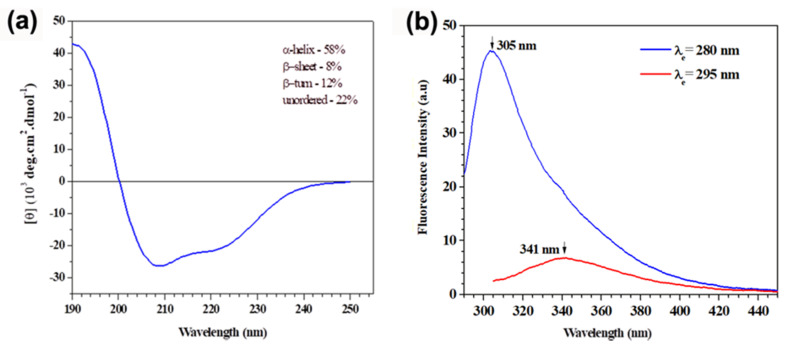
Spectroscopic characteristics of the AaCI-2S. Samples contained a 3 µM concentration of inhibitor, in PBA buffer 10 mM, pH 7.0. (**a**) Far UV-CD spectrum was recorded using a 1 mm cell path length cylindrical cuvette with an average of 8 scans, at 25 °C. The CDPro program was used to estimate the AaCI-2S secondary structure. (**b**) Fluorescence emission spectra of AaCI-2S. The samples were excited at 280 nm and 295 nm, and the fluorescence emission was monitored in the 290–450 and 305–450 nm ranges, respectively, at 25 °C.

Circular dichroism (CD) spectroscopy was used to characterize the secondary structure of the inhibitory molecule. The spectrum displayed two negative bands, one at 208 and another at 222 nm, a positive band at 192 nm (Figure 3a), and its deconvolution estimated 58% of α-helix, 12% of β-turns, 8% of β-sheets, and 22% of disordered structures. The cluster analysis indicated that AaCI-2S belonged to the α+β class of proteins, which presented a more pronounced band at 208 nm than the one at 222 nm [17], the typical secondary structure of members of the prolamin superfamily [18], similar to napin [19] and 2S albumin isolated from melon *Momordica charantia* [20].

The emission fluorescence measurements of the AaCI-2S aromatic amino acids with excitation wavelengths at 280 nm (blue line) and 295 nm (red line) are shown in Figure 3b. The recorded spectra were very different in both the form and location of the emission peak. The intrinsic fluorescence analysis exhibited that with an excitation at 295 nm, the emission peak occurred at 341 nm, which is a characteristic profile of tryptophan class II residues partially exposed to solvent, as in sunflower [21] and buckwheat *Fagopyrum esculentum* 2S albumins [22].

### 2.4. Effects of pH and Temperature on the Activity and Structure of the AaCI-2S Inhibitor

**Figure 4:**
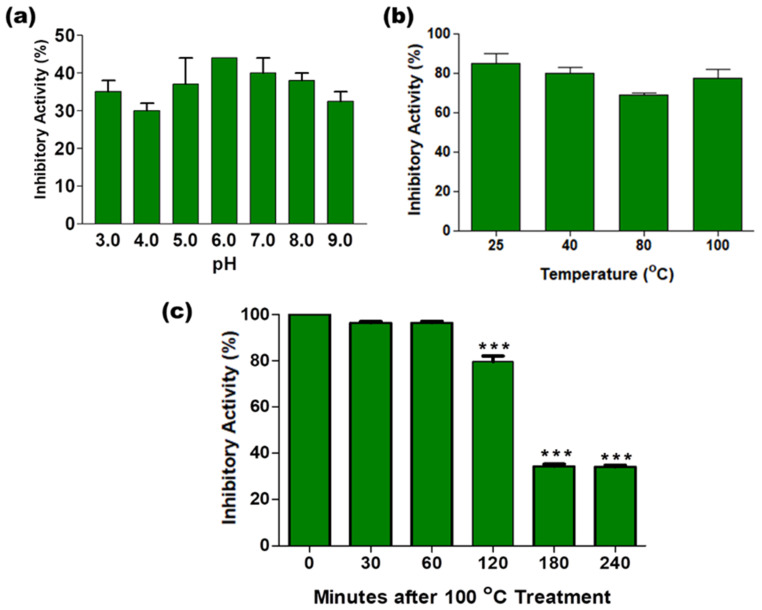
Effects of pH and temperature on the activity of the AaCI-2S inhibitor. (**a**) Functional stability at different pH values. The inhibitor samples were pre-incubated in solutions with different pH values for 30 min, neutralized to an initial pH (8.0), and the inhibitory activity on papain assay was measured. (**b**) Functional stability at different temperatures. The inhibitor was heated at different temperatures for 30 min. (**c**) Functional stability at 100 °C for up to 4 h. After boiling, the inhibitory activity on papain was measured. ((**b**,**c**) after different pretreatment temperatures, the samples were cooled down at room temperature for 30 min before the inhibitory assays). (*** *p* < 0.0001, one-way ANOVA, follow Tukey’s multiple comparison test).

**Figure 5:**
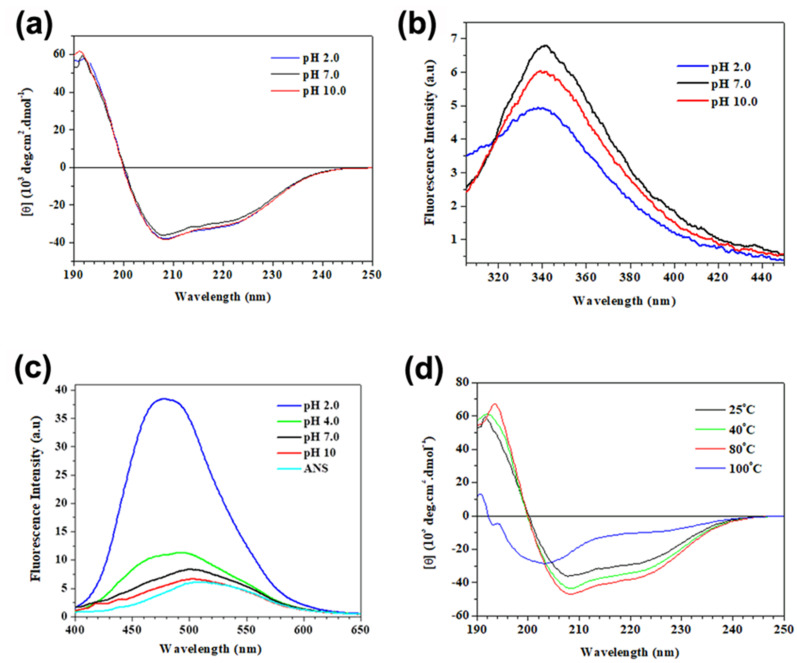
Effects of pH and temperature on AaCI-2S conformation. For pH dependence assays, the inhibitor (4 µM) was incubated in 10 mM PBA buffer for 30 min, at 25 °C: (**a**) Far-UV CD spectra, (**b**) Tryptophan fluorescence spectra of the AaCI-2S at different pH values (blue line, pH 2.0; black line, pH 7.0 and red line, pH 10.0) (**c**) ANS fluorescence spectra in the absence (cyan line) and presence of AaCI-2S as a function of pH. Spectra were taken 30 min after the addition of ANS probe to the protein samples. (**d**) Temperature effects on CD spectra of AaCI-2S (4 µM), in 10 mM PBA buffer, pH 7.0. Before measurements, samples were incubated at their respective temperatures for 30 min and then cooled to 25 °C.

AaCI-2S was stable over a wide pH range (Figure 4a) and temperature (Figure 4b). Its stability was confirmed by CD spectroscopy since no modifications of its secondary structure were observed in the pH range of 2 to 10 (Figure 5a). The results of the intrinsic fluorescence emission of this inhibitor revealed that the microenvironment of Trp residues also did not undergo significant changes in this pH range (Figure 5b), maintaining the emission peak at around 341 nm and subtle variations in intensity. However, as these residues are already partially exposed to the solvent, the ANS extrinsic probe was used to monitor the global conformational changes in the tertiary structure of the inhibitor. This dye has a low fluorescence quantum yield in aqueous environments because it binds preferentially to the hydrophobic sites, promoting a pronounced increase in the fluorescence intensity and a blue shift of the emission peak. Figure 5c showed that significant changes in the fluorescent properties of the probe occur only in acidic environments since the emission peak maximum shifts toward shorter wavelengths (from 510 nm to 478 nm) and the fluorescence intensity increases up to seven-fold, at pH 2, suggesting the exposure of hydrophobic regions at this pH, which was previously inaccessible to the probe. The thermal stability of the secondary structure of the inhibitor can be monitored by the decrease in the CD bands at 208 and 222 nm (Figure 5d). Partial loss of structure was observed after treatment at 100 °C, wherein the inhibitor lost 20% of its activity within 2 h (Figure 4b), but it did not disappear completely even after 3 or 4 h of incubation (Figure 4c).

### 2.5. Inhibitor Sequence

**Figure 6:**
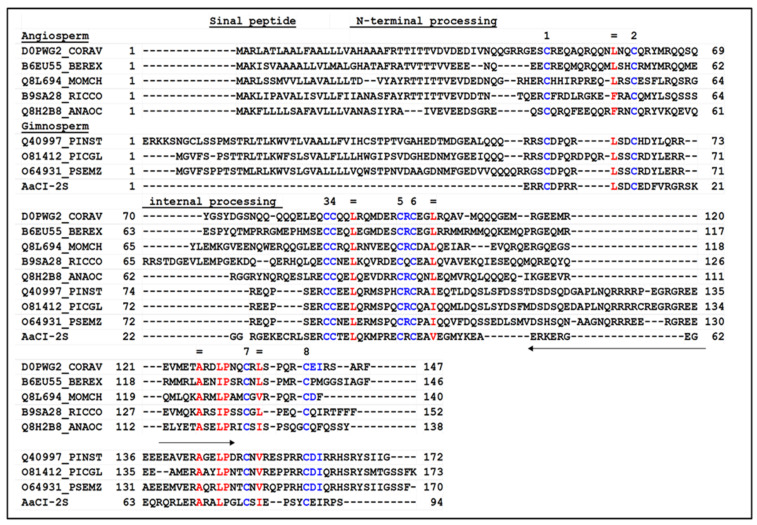
Comparison between the amino acid sequence obtained from AaCI-2S with precursors of 2S reserve proteins from angiosperms and gymnosperms. The sequences were aligned using ClustaW2. Similar strings include D0PWG2_CORAV: hazelnut (*Corylus avellana*); B6EU55_BEREX: Brazil nut (*Bertollethia excelsa*); Q8L694_MOMCH: São Caetano melon (*Momordica charantia*), B9SA28_RICCO: castor bean (*Ricinus communis*); Q8H2B8_ANAOC: cashew nut (*Anacardium occidentale*); Q40997_PINST: pinus (*Pinus strobus*); O81412_PICGL: spruce (*Picea glauca*); and O64931PSEMZ: Douglas fir (*Pseudotsuga menziesii*). Spaces (-) were introduced to maintain alignment. The eight conserved cysteine residues are indicated numerically in blue. The conserved hydrophobic residues are in red, and the arrows indicate a region rich in arginine and glutamic acid residues.

The amino acid sequence of the inhibitor when compared with other protein sequences in the UniProt Knowledgebase database reveals similarities with 2S albumins of conifers and angiosperms. Because of the inhibitory activity on cysteine proteases and the structural similarity with 2S-albumin, the isolated *A. angustifolia* inhibitor was named AaCI-2S. Figure 6 showed the multiple alignments of AaCl-2S with conifer and angiosperm 2S albumin. The highest scores were obtained with conifers *Pinus strobus*, *Picea glauca*, and *Pseudotsuga menziesii* and with the angiosperms *Corylus avellana* (hazel) and *Anacardium occidentale* (cashew tree). A comparison of the AaCl-2S sequence was also performed using the BLAST program with those deposited in the MEROPS database of peptidases and their inhibitors. The search revealed a similar identity with a family of proteins whose inhibitory activity has not yet been demonstrated, denominated in the database by “Family I6 unassigned peptidase inhibitor homolog.” The protein sequence data reported in this paper will appear in the UniProt Knowledgebase under the accession number C0HLT8.

### 2.6. Biological Properties of AaCl-2S

**Figure 7:**
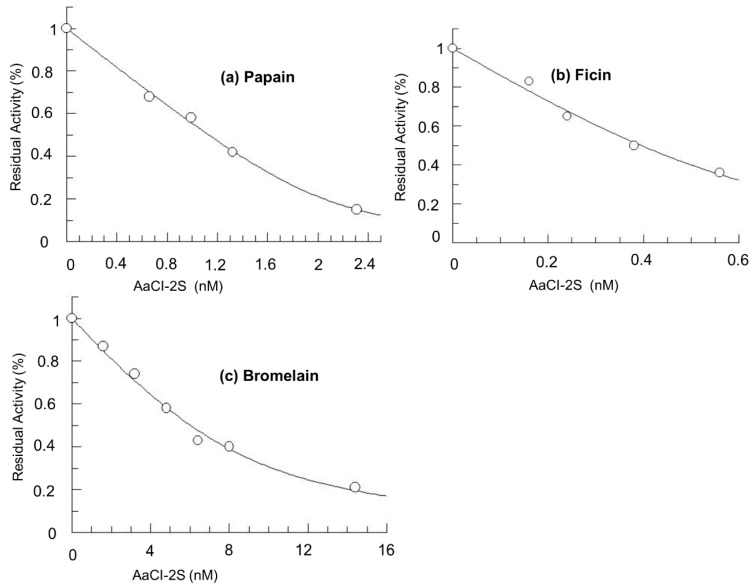
AaCI-2S inhibitory properties. Increasing the concentration of *Araucaria angustifolia* cysteine protease inhibitor was incubated with (**a**) papain (2 nM), (**b**) ficin, and (**c**) bromelain for 20 min, at 40 °C in 0.1 M Na_2_PO_4_ buffer (pH 6.3) containing 0.4 M NaCl, 0.01 M EDTA, and 8 mM DTT, the enzymatic activities were determined by the hydrolysis of Z-Phe-Arg-pNan (5 mM).

The inhibition curves of some cysteine proteases by AaCl-2S was reported in Figure 7. The calculated K_iapp_ of the inhibition of cathepsin L (K_iapp_ = 0.01 nM) was about 20 times lower than that of papain inhibition (K_iapp_ = 0.2 nM), which reflects a greater affinity for cathepsin L. AaCI-2S presented no inhibitory activity on cathepsin B, but it also inhibited other enzymes in the papain family, such as ficin (K_iapp_ = 1.1 nM) and bromelain (K_iapp_ = 8.4 nM). No inhibitory activity was detected on serine proteases (trypsin, human plasma kallikrein, and elastase).

### 2.7. Effect of AaCI-2S on Predatory Insect Enzymes

**Figure 8:**
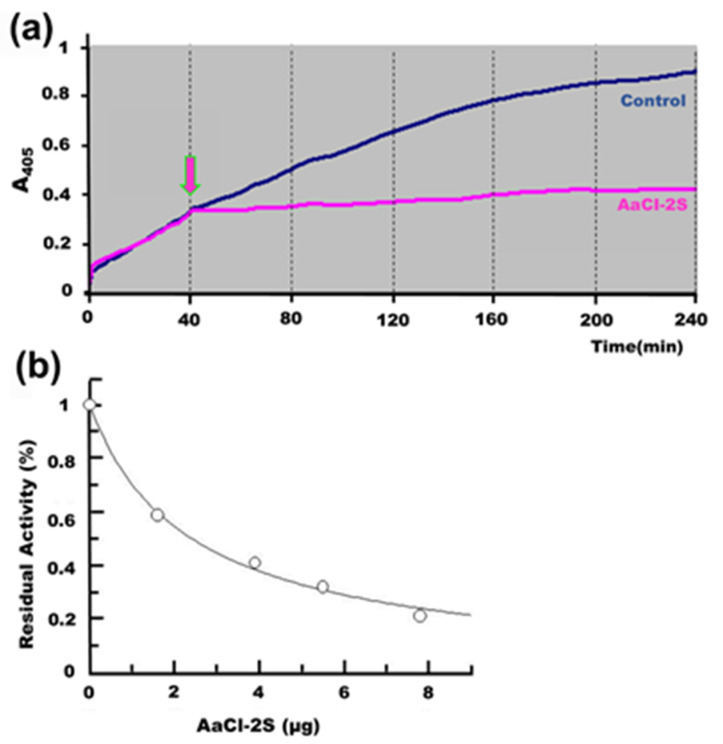
Action of AaCI-2S on the proteolytic activity of *Callosobruchus maculatus* larvae. (**a**) The blue line indicates the increase of proteolytic activity on Z-Phe-Arg-pNan. of the medium intestinal extract containing 43 µg of total proteins. The arrow indicates the time of the addition of 10 μg of the inhibitor to the incubation medium. The pink line indicates a decrease in proteolytic activity. (**b**) Inhibition of the proteolytic activity extracted from the intestine of *Callosobruchus maculatus*. A medium intestinal extract containing 11.5 μg of proteins was preincubated at 37 °C for 10 min with increasing concentrations of AaCl-2S in 0.1 M Na_2_PO_4_ buffer at pH 6.3, 0.4 M NaCl, 01 M, and 8 mM DTT. Residual activity was determined by the hydrolysis of Z-Phe-Arg-pNan (5 mM).

Enzymes of the class of cysteine proteases are the major proteolytic enzymes of coleopteran larvae. One of the physiological reasons for the presence of proteins with inhibitory activity in plant seeds is their involvement in the mechanism of seed protection against predatory insects. For this reason, we used larvae from the cowpea bruchid, *Callosobruchus maculatus,* a predator of string bean seeds *Vigna unguiculata*, as a model to investigate whether purified AaCI-2S would decrease the proteolytic activity in the midgut extract of these larvae. Figure 8a indicated the rapid interaction of the inhibitor added to the incubation medium after 40 min and the resulting decrease in proteolytic activity on the colorimetric substrate Z-Phe-Arg-pNan. Figure 8b showed the inhibitory effect of increasing the concentrations of AaCl-2S on the residual activity of cysteine proteases present in the midgut of larvae.

### 2.8. Investigation on the Antitumor Activity of AaCI-2S 

Pine nuts are used by man as a functional food and, since cysteine proteases are involved in several types of tumors, we were interested in investigating the effect of AaCI-2S on tumor cells, where cathepsin L is recognized to play an important role, as in the models of gastric cancer and prostate cancer.

**Figure 9:**
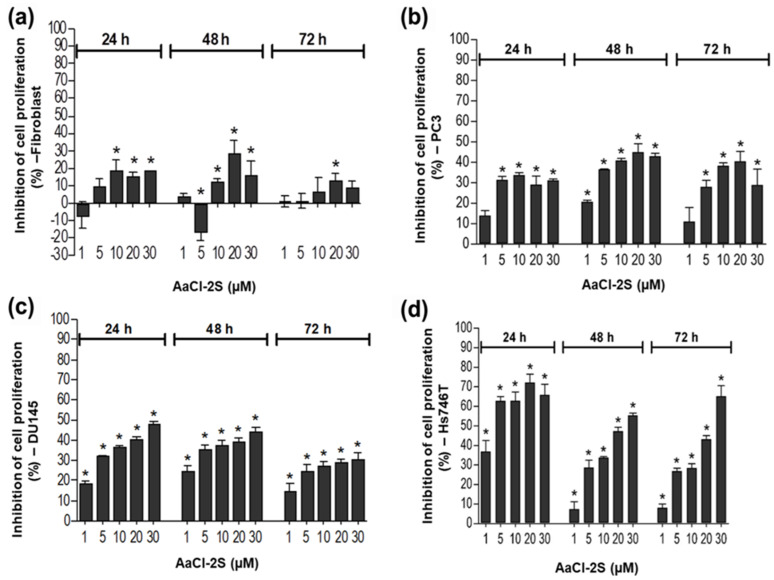
Effects of the inhibitor on the proliferation of prostate cancer cells, gastric cancer, and human fibroblasts. Effect of AaCl-2S on the proliferation of (**a**) fibroblasts, (**b**) PC3, (**c**) DU145, and (**d**) Hs746T cells. Cells were pre-incubated with increasing concentrations of AaCl-2S for 15 min at room temperature and analyzed at different incubation times (* *p* < 0.05, unpaired *t*-test).

The effects of the inhibitor on the proliferation of prostate cancer cells (DU-145 and PC3), gastric cancer (Hs746T), and non-tumor human fibroblasts were illustrated in Figure 9. The inhibitor did not affect fibroblast (a) proliferation, while it inhibited the proliferation of both prostate cancer cell lines PC3 (b), DU-145 cells (c), and of the Hs746T cells (d).

## 3. Discussion

In angiosperms, a considerable fraction of seed proteins includes inhibitors of serine proteases; however, to date, not many protease inhibitors have been purified and characterized in gymnosperms [23]. This was also confirmed by our investigation since, in the present study, the saline extract of *A. angustifolia* seeds did not inhibit trypsin or other serine proteases. A trypsin inhibition has been detected in the embryo tissues only after sample concentration with acetone precipitation [24]. In contrast, the saline extract inhibited two cysteine proteases, papain, and the enzyme cruzain (a recombinant form of the cysteine protease cruzipain from *Trypanosoma cruzi*) [25]. 

To our knowledge, there are no reports on the effects of cysteine protease inhibitors against termites. However, the deleterious effects found in termites have been attributed to lectins and serine protease inhibitors [16,26,27,28] and this might not be the case of pine nuts. Although further studies are needed, the description of the termiticidal potential of the seeds is relevant, as it shows that nature has selected an alternative to its century-old forest protection against this pest. This property can be exploited commercially as an alternative for the use of Araucária other than its wood, thus protecting the forest, a world heritage site.

The presence of papain inhibitors has been reported in seeds of some gymnosperms such as *Pinus maritima*, *Picea pungens*, and *A. angustifolia* [29]; however, purification and characterization of the inhibitory activity were not achieved. We did not find any purified gymnosperm phytocystatin in the protein databases, but approximately 200 sequences have been identified through comparative genomic analysis [30]. These analyses have been largely useful for information on the conservation and evolution of proteolytic enzymes and their inhibitors [31].

The concentration of the inhibitor, determined by titration with papain, was 17 mg/kg. The first cysteine protease inhibitor (oryzacystatin) identified in rice during the 1980s occurs at a concentration of 2–3 mg/kg [32] and its structural and functional characterization was investigated only after its recombinant form was reported [33]. In contrast, the concentration of trypsin inhibitors is rather high in legumes [34], for example, the concentration in *Enterolobium contortisiliquum* is approximately 5600 mg/kg [35].

The fact that the inhibitor does not bind to ion exchange resins is an alternative and effective method for use in the processing of large amounts of extract through the batchwise system. Using this strategy, most of the proteins with a molecular weight above 25 kDa were eliminated, thereby favoring size exclusion chromatography in Superdex 30, in which the inhibitory activity was detected only in the second peak. Structurally, the protein database sequences displayed high similarity with conserved proteins of the 2S albumin family and no sequence homology with the typical phytocystatins or with other plant inhibitors of cysteine proteases as the described inhibitor of *B. bauhinioides*, BbCI, which also differs from phytocystatins [36]. 

From the earliest studies with protease inhibitors in plants, their potential role as a reserve protein has been postulated. The similarity with the 2S albumins of the gymnosperms *Picea glauca*, *Pseudotsuga menziesii* [37], and *Pinus strobus* was 64%, 55%, and 52.6%, respectively. Similarities of 62.5% and 67.5% were also observed with some 2S angiosperm albumins such as *Corylus hazelnut* [38] and *Anacardium occidentale* (cashew nuts) [39], respectively. Thus, based on similarities, sequence alignment, and arrangement of conserved cysteine residues, we can conclude that the *Araucaria angustifolia* inhibitor is a reserve protein of the 2S albumins class which justified the denomination adopted in this work (AaCI-2S). The AaCI-2S also displayed a high content of arginine residues, which is a common feature in conifer reserve proteins. Furthermore, 8 cysteine residues and the hydrophobic residues flanking cysteine residues are conserved positions in relation to the 2S gymnosperm and angiosperm albumins as in the sequence of 2S albumin from *Pseudotsuga menziesii* [37]. Additionally, AaCI-2S displays a molecular mass of 18 kDa and two identical polypeptide chains linked by disulfide bonds similar to the storage protein 2S albumin. Although the reserve function of proteins is usually assigned to 2S albumins, other biological activities have been described such as inhibiting fungal growth [20], hemagglutinating activity [40], the inhibitory activity of trypsin [41], and in the case of AaCI-2S, the inhibitory activity of cysteine proteases. 

Many larvae of insects of the order Coleoptera are predators of seeds and the presence of cysteine proteases as digestive enzymes [42] leads to the hypothesis of a possible exogenous protective role of inhibitor of this class of enzymes. Numerous studies evidencing the in vitro and in vivo inhibition of the digestive proteases of these larvae [43,44] and on the growth of fungi [45] support this role. The possible action of the inhibitor to act as a defense protein was confirmed by the rapid and effective inhibition of the cysteine protease activity present in the insect intestine suggesting that this inhibitor may be employed in the functional study of these enzymes. Naturally, these insects do not use the seeds of *Araucaria*, but the result is interesting in the sense of indicating how proteins can be strategic in the composition of compounds involved in the protection of seeds in general. 

The presence of protease inhibitors in legume seeds and cereals added to epidemiological studies that identify legumes as potential protective agents in reducing the incidence of some types of cancer in the vegetarian population have stimulated a series of studies involving inhibitory proteases influencing tumor promotion in vivo and in vitro [46,47,48].

In addition to the inhibition of cruzain and papain, AaCI-2S seems to be selective regarding the two human cathepsins tested, since cathepsin L was inhibited while cathepsin B activity was not altered. Both are medically relevant targets because they are involved in many physiological and pathological processes such as apoptosis, inflammation, and cancer [49]. Among other functions, cathepsins (mainly cathepsins B and L) are involved in extracellular matrix degradation, facilitating the growth, invasion, and metastasis of tumor cells [50,51]. AaCl-2S can interfere with the cellular proliferation of the two cell lines of prostate cancer and shows a more effective inhibitory effect on the proliferation of gastric tumor cells. Increased activity of cathepsins B and L and the reduction of secreted endogenous cystatins have been observed in prostate cancer cell lines PC3 and DU145. The invasive ability of these cell lines was partially inhibited by E-64, a synthetic inhibitor of cysteine proteases [52]. It is worth mentioning that the non-interference in non-tumorigenic cells demonstrate the inhibitory selectivity in cancer cell lines. As the seeds of *Araucaria* are used as food, their antiproliferative effect is of nutritional significance for future studies and provides important information regarding the benefits of including the pine nut in our diet.

The overall yield of the inhibitor by the purification process was low (12%), thus obtaining large amounts of inhibitor is difficult. Notably, this loss is not due to thermal stability, since the inhibitor spectra following treatment at different temperatures exhibited a structure that was quite resistant to thermal denaturation, displaying small conformational changes after heating up to 80 °C. Only the treatment at 100 °C caused a modification of the inhibitor structure with a partial loss of structure. These results are consistent with other observations regarding the thermostability of many members of the prolamin superfamily, such as the presence of intramolecular disulfide bonds, which have been implicated in this thermostability [53]. The maintenance of the inhibitory activity against papain after heating and exposure to extreme pH values indicates that the inhibitor is functionally stable. Many other protease inhibitors purified from plant seeds exhibit high stability at various temperatures [54], but we were surprised that the inhibitor isolated from the *Araucaria* seed maintained its activity even after a 60 min treatment at 100 °C and may implicate in the qualification of pine nuts as a functional food since they are normally consumed roasted or cooked.

## 4. Material and Methods

### 4.1. Plant Material

*Araucaria angustifolia* (Bertol.) O. Kuntze plant material was deposited in the Herbarium of Universidade Estadual da Bahia, UEDB, identified as HUESB 12431. The studies were conducted in accordance with Brazilian legislation (license no. 02/2014, process 02000.003472/2005– 62 Ministério do Meio Ambiente, Coordenação Geral de Autorização de uso da Flora e Floresta, SCEN).

The seeds were purchased from the city of Campos do Jordão—SP from the natural occurrence of *A. angustifolia* located in Campos do Jordão State Park.

### 4.2. Experimental Reagents

Bovine serum albumin, fibronectin, human neutrophil elastase (EC 3.4.2.37), and porcine pancreas elastase (EC 3.4.21.7) were purchased from Calbiochem^®^—(EMD Chemicals Inc., Port Wentworth, GA, USA). Bovine trypsin (EC 3.4.21.4), papain (EC 3.4.22.2), bromelain (EC 3.4.22.32), and ficin (3.4.22.3) were obtained from Sigma-Aldrich (Co., St. Louis, MI, USA). Kallikrein (human plasma) (EC 3.4.21.34) was purified according to Oliva [55]. Cruzain, cathepsin B, and L were provided by Prof. Dr. Luís Juliano Neto, Department of Biophysics, UNIFESP. Chromogenic substrates derived from p-nitroanilide (Bz-Arg-pNan, HD-Pro-Phe-Arg-pNan, Suc-Phe-pNan, HD-Val-Leu-Lys-pNan, HD-Phe-L-Pip-L -Arg-pNan, MeO-Suc-Ala-Ala-Pro-Val-pNan, N-Suc-Ala-Ala-Pro-Phe-pNan), and the fluorimetric aminomethyl coumarin substrate Z-Phe-Arg AMC were obtained from Calbiochem^®^ (EMD Chemicals Inc., USA), and starch from Sigma-Aldrich Co. (Saint Louis, MO, USA). 

DEAE–Sephadex^®^ A-50; SP-Sephadex^®^ C-50 e Superdex^®^ 30 (GE Healthcare, Chicago, IL, USA)—Biogel^®^ P30 (Bio-Rad Laboratories, Hercules, CA, USA)—C18 column *Protein & Peptide (Vydac*^®^ *Ultrasphere*—Brea, CA, USA)—Column *Aquapore*^®^ *RP 300 C* (Varian, Palo Alto, CA, USA). Dinitrosalicylic acid (ADNS), ammonium persulfate, MTT salt, toluidine blue dye, E-64, and 1-anilinonaphthalene-8-sulfonic acid (ANS) probe were obtained from Sigma-Aldrich Co. (USA). Coomassie Brilliant Blue R-250 was obtained from Bio-Rad Laboratories (USA). Fetal bovine serum LB broth, cell culture media, RPMI 1640, DMEM, TEMED, and dithiothreitol from Gibco Invitrogen Co. (Waltham, MA, USA). Acrylamide and N, N, and methylene bisacrylamide were obtained from Serva (Heidelberg, Germany). Molecular weight standards were obtained from Fermentas Inc. (Burlington, ON, Canada) and Bio-Rad Laboratories (USA).

### 4.3. Cells

The PC3 cell line of prostate adenocarcinoma and the cell line HsT46T of gastric adenocarcinoma were provided by Prof. Dr. Barbara Mayer, from the Klinikum Groβhadern Surgery Department, University of Munich, Germany. The DU145 prostate adenocarcinoma cell line was provided by Prof. Dr. Heloisa Selistre de Araújo, from the Department of Physiological Sciences at the Federal University of São Carlos. The human lineage of fibroblasts, obtained from cells of the amniotic fluid, was provided by Prof. Dr. Leny Toma, from the Biochemistry Department at the Federal University of São Paulo.

### 4.4. Protein Extraction and Fractionation

The seeds were ground in a blender with a 0.15 M NaCl solution, at a 10% (*w/v*) density, heated at 60 °C for 30 min, cooled in an ice bath for 30 min, stirred at room temperature for 20 min, filtered with cotton and gauze, and centrifuged at 6000× *g* for 15 min at 4 °C. Proteins were estimated spectrophotometrically (A280) as well as by Bradford (1976) [56] assay using bovine serum albumin as the standard.

### 4.5. Inhibitory Activity

The protein extract of plant seeds as well as the purified inhibitor was tested on proteases. The p-nitroaniline released as a hydrolysis product was measured at 405 nm using a SpectraCount spectrophotometer. In the case of the fluorogenic substrate Z-Phe-Arg-AMC, the Hitachi F-2000 spectrofluorometer was used with excitation and emission wavelengths of 380 and 460 nm, respectively. Different concentrations of the inhibitor solutions were added to the appropriate volumes of activated enzymes in a 100-μL volume of buffer. The volume was topped to 230 µL with a 0.15 M NaCl solution and the mixture was pre-incubated at 37–40 °C for 10 min before the addition of the substrate. The reaction proceeded for 20–30 min at 37–40 °C and was stopped by the addition of 40% acetic acid (*v/v*). The absorbance obtained in the absence of the extracts was considered as 100% of enzymatic activity, and the inhibition was expressed as the reduction of enzyme activity percentage [57].

The concentration of active papain was determined by titration with the synthetic inhibitor E-64 according to Zucker et al. [58]. Once the inhibitory activity of papain was determined, the purified protein was titrated and used to determine the dissociation constants of the enzyme/inhibitor complexes (K_iapp_) with other cysteine proteases. The determinations were performed following the model suggested by Morrison adapted to an enzymatic kinetics program for computer graphics, and the numerical value was calculated using the GraFit program [59].

### 4.6. Evaluation of the Extract Effects on Adult Insect Survival

The insecticidal activity of the extract was evaluated by a bioassay based on the method described by Kang [60]. Each assay consisted of a Petri dish (90 × 15 mm) with the bottom covered by a paper filter. Disks (4 cm in diameter) of paper filter were impregnated with 200 µL of extract (0.2, 0.4, 0.8, or 1.0 mg/mL). In the negative control, 200 µL of 0.15 M NaCl was added to the disks. A total of 20 active termites (at a worker-to-soldier ratio of 4:1) were transferred to each dish, which was maintained at 28 °C in the dark. Insect survival was evaluated daily until the death of all insects. The bioassays were carried out in quadruplicate for each tested concentration, and the survival rates (%) were calculated.

### 4.7. Inhibitor Purification by Ion-Exchange Chromatography (Batchwise)

The proteins from seed extract were precipitated by the slow addition of ice-cold acetone to a final concentration of 80% (*v/v*). After a sedimentation period (30 min), part of the acetone was sucked out with a rubber cannula, and the remaining fraction was centrifuged at 3000× *g* for 15 min at 4 °C. The acetone-precipitated proteins were spread on Petri dishes, dried at 24 °C, and then frozen until use. The acetone-precipitated protein was dissolved in water (1 g/10 mL), centrifuged at 3000× *g* at 4 °C for 15 min, and the conductivity values of the solutions were adjusted by 0.05 M Tris-HCl (pH 8.0) buffer using DEAE–Sephadex^®^, ion-exchange chromatography. The protein was added to the resins and stirred for 30 min. The mixture was filtered through a funnel with a porous plate, and the resin was washed with an equilibration buffer to remove the non-adsorbed proteins. Elution was performed using 0.15 M or 0.3 M NaCl solution in the equilibration buffer. The non-adsorbed fraction containing papain inhibitory activity was mixed with the SP-Sephadex resin and the procedure was repeated as described above. Papain inhibitory activity was also detected in the non-adsorbed fraction.

### 4.8. Molecular Exclusion Chromatography

The non-retained ion exchanging fraction was dialyzed in water using a 10 kDa cut off membrane, lyophilized and dissolved in a 0.05 M Tris-HCl buffer (pH 8.0) containing 0.15 M of NaCl, centrifuged at 10,000× *g* for 5 min at room temperature, and loaded onto a Superdex 30 column in a 0.05 M Tris-HCl buffer (pH 8.0) containing a 0.15 M NaCl (equilibrium buffer) under a 0.5 mL/min flow rate in the ÄKTA purifier system (GE Healthcare). The molecular mass of the inhibitor was estimated by standardizing the column with ferritin (440 kDa), SBTI (20 kDa), cytochrome C (12.4 kDa), and aprotinin (6.5 kDa). The protein profile was monitored by absorbance at 280 nm, and fractions (1 mL) with inhibitory activity were pooled, dialyzed, and lyophilized.

### 4.9. Reverse-Phase Chromatography on an HPLC System

The protein from Superdex 30 chromatography was further purified by C18 reverse phase (Protein & Peptide, 4.6 mm × 14 cm) in an HPLC system (Model SCL-6A—Shimadzu), equilibrated with a 0.1% trifluoroacetic acid (TFA) solution in water (Solvent A). The elution was performed on a gradient of 0.1% TFA in water with 90% acetonitrile (Solvent B) under a constant flow rate of 0.7 mL/min [61].

### 4.10. Sodium Dodecyl Sulfate-Polyacrylamide Gel Electrophoresis

Denaturing electrophoresis was performed according to the method described by Laemmli [62] on a polyacrylamide gel (15%) in the presence of SDS. The samples were treated with a reducing agent in dithiothreitol-containing sample buffer (200 mg/mL) and heated for 10 min at 100 °C. The proteins were visualized by staining with Coomassie blue R250 solution.

### 4.11. Estimation of Secondary Structure by Circular Dichroism (CD) Spectroscopy

The far-ultraviolet (UV) CD spectra of the purified inhibitor (an average of 8 scans) were recorded on a J-810 (Jasco Corporation, Tokyo, Japan) spectropolarimeter within the range of 190 to 250 nm in a 1-mm optical path cylindrical quartz cuvette at 25 °C. The inhibitor (3 µM) was dissolved in 10 mM PBA buffer (pH 7.0) and its CD spectra were expressed as molar ellipticity [θ]. The estimated calculation of the secondary structure fractions was performed using the CDPro deconvolution package, with the Selcon3, Continll, and CDSSTR programs [63].

### 4.12. Intrinsic Fluorescence Measurements

The fluorescence emission measurements were obtained using an F-2500 fluorometer (Hitachi Ltd., Tokyo, Japan) at 25 °C in quartz cuvettes, with an optical path of 1 cm. Analyses were performed with the purified inhibitor (3 µM) in 10 mM PBA buffer (pH 7.0). The sample was excited at 280 or 295 nm, and the fluorescence emission was monitored in the range of 290–450 and 305–450 nm, respectively. The fluorescence emission spectra of buffers were subtracted from the spectra of the samples to minimize the effect of light scattering and to perform baseline corrections [64].

### 4.13. Studies on the Influence of pH and Temperature on Structural Stability

Purified inhibitor structural stability was analyzed under different conditions through the measurements of CD and intrinsic and extrinsic fluorescence emission. Samples of the inhibitor (4 µM) in 10 mM PBA buffer (pH 7.0) were incubated at 25, 40, 80, and 100 °C for 30 min and then cooled. Samples with the same concentration were incubated in solutions of different pH (pH 2, pH 4, pH 7, and pH 10) for 30 min. After each treatment, the measurements of CD and intrinsic fluorescence were performed under the same conditions described above. For the extrinsic fluorescence measurements, the treated samples were incubated with the 8-anilino-1-naphthalenesulfonate probe (ANS), 90 µM in 10 mM PBA buffer, pH 7.0 for 15 min, at 25 °C, and the fluorescence spectra were recorded at 400 to 650 nm with a 385 nm excitation, 30 min after adding the probe.

### 4.14. N-Terminal Sequence Determination

After reverse phase chromatography, the inhibitor (2 nM) was dissolved in 300 μL of buffer 0.25 M Tris-HCl (pH 8.5) containing 6 M guanidine, 1 mM EDTA, and 5 μL of β-mercaptoethanol and then incubated for 2 h at 37 °C, under a nitrogen-saturated atmosphere in the absence of light. Alkylation was performed with the addition of 5 mL of vinylpyridine and re-incubation for 90 min at 37 °C and subjected to HPLC/acetonitrile/isopropanol reverse phase chromatography at a constant flow rate (0.1 mL/min). The N-terminal sequence was obtained automatically by the Edman (1949) [65] degradation method in two independent laboratories. Similarity searches were performed using the FASTA program using the *UniProt Knowledgebase* database Larkin [66] and a *Blossom 80* (EBI) [67] matrix (www.ebi.ac.uk). Multiple alignments of similar sequences were performed using the ClustalW2 program (http://www.ebi.ac.uk/Tools/clustalw2/).

### 4.15. Effect of pH and Temperature on Inhibitor Activity

To verify the effect of pH, the inhibitor (1 µg) was pre-incubated for 30 min in 50 mM sodium citrate buffer (pH 3 and pH 6) or 50 mM Tris-HCl buffer (pH 7 and pH 9). After pre-incubation, the pH of the samples was adjusted to 8.0 with Tris-HCl, and the ability to inhibit papain was determined. The lability of the inhibitor at different temperatures was investigated by incubating the samples (1 µg) in 50 mM Tris-HCl pH 8.0 buffer, keeping them in a water bath at different temperatures (25, 40, 80, and 100 °C) for 30 min. The resistance of the inhibitor to boiling (100 °C) was also studied by incubation for different durations (30 min, 1, 2, 3, and 4 h). After different heat treatments, the samples were cooled in an ice bath for 5 min and tested for their inhibitory activity.

### 4.16. Evaluation of the Inhibitor Effect on Insect Enzymes

Twenty larvae of *C. maculatus* with 19–20 days post-hatching (4th instar) were removed from the infested seeds and immersed in 0.15 M NaCl solution for dissection of the intestines with the help of watchmaker tweezers and stereoscopic magnifying glass. Lysis was performed in the intestines by brief sonication wells at 150 µL solution NaCl 0.15 M. The obtained extract was centrifuged at 10,000× *g* at 4 °C for 10 min. The supernatant was collected and frozen at −20 °C. In a preliminary test, 50 µL of crude extract diluted ten times and containing 12 µg of protein was added in duplicates into two wells of a microplate with Na_2_PO_4_ 0.1 M buffer (pH 6.3), 10 mM EDTA, and 0.4 M NaCl. The substrate (20 μL) Z-Phe-Arg-pNan (0.05 M) was added in a final volume of 250 μL. The plate was incubated at 37 °C and hydrolysis was followed photometrically for 40 min. Next, the purified inhibitor (10 μg) was added to one well, and substrate hydrolysis continued to be monitored for up to 4 h. To evaluate the effect of inhibitor concentrations, the extract diluted ten-fold in 0.1 M Na_2_ PO_4_ buffer (pH 6.3), 10 mM EDTA; 0.4 M NaCl; 8 mM DTT remained at 37 °C for 10 min for enzymatic activation. Next, 50 μL of the activated extract containing 11.5 µg of proteins were preincubated in the absence and presence of different inhibitor concentrations (1 to 8 ug) for 10 min at 37 °C. After the addition of 20 μL of the Z-Phe-Arg-pNan substrate (5 mM) in a final volume of 250 μL, the hydrolysis was monitored for 60 min and then quenched with 50 μL of 40% acetic acid (*v/v*).

### 4.17. Studies on the Effect of Inhibitors on Tumor Cells and Human Fibroblast Cells

PC3, DU145, and Hs746T tumor cell lines were maintained in RPMI-1640 culture medium and the fibroblast cells were maintained in Dulbecco’s modified Eagle’s medium (DMEM), pH 7.4. Both culture media were enriched with 10% fetal bovine serum, penicillin (10 UI/mL), and 100 µg/mL streptomycin. Cells were sub-cultured weekly using the following protocol: The medium was removed from the confluent cell flasks (60 × 10 mm) and cells were washed with PBS solution (pH 7.4). For cell detachment, the cells were incubated with 1 mL trypsin solution (0.25%) for 1 min. Next, 1 × 10^5^ cells were resuspended, transferred to a new plate in the appropriate media, cultured at 37 °C under 5% CO_2_, and the culture medium was changed every 3 days [46,47].

### 4.18. Cell Viability Assay

PC3, DU145, and Hs746T cells (5 × 10^3^ cells/100 μL/well) and fibroblast cells (8 × 10^3^ cells/100 μL/well) were incubated at 37 °C and 5% CO_2_ for 24 h in RPMI-1640 medium containing 10% fetal bovine serum. A total of 100 μL of inhibitor (2.5–30 μM) diluted in RPMI-1640 medium, previously filtered through a Millipore filter (0.22 μm), was added to the adhered cells and incubated for 24, 48, and 72 h. At the end of each incubation period, 10 μL of MTT (tetrazolium salts) dissolved in PBS (5 mg/mL) was added to each well and the cells were again incubated for 2 h. Subsequently, the medium was removed and 100% DMSO was added to solubilize the formazan crystals and incubated for 20 min at 37 °C. The absorbance was measured at 540 nm using a spectrophotometer (SpectraCount model). Assays were performed in triplicate for each inhibitor concentration and experiments were performed twice as described by Gasperazzo Ferreira et al. [68].

### 4.19. Statistical Analyses

All assays were performed in triplicate and independently. The statistical analyses were expressed as the mean ± standard deviation (SD) and analyzed using GraphPad Prisma Software. Comparisons among the variables, measured in defined experimental groups, were conducted using one-way ANOVA, followed by Tukey’s test. Statistical significance was defined as * *p* < 0.05, ** *p* < 0.005, and *** *p* < 0.0001.

## 5. Conclusions

These findings provided relevant information about the insecticide and antitumor activity of the pine nuts, the potential biotechnology application in agriculture, and human health that can contribute to the preservation of the Araucária forest. Also, a protein named AaCI-2S, with a molecular mass of 18 kDa composed of two identical polypeptide chains linked by two disulfide bonds was characterized. The studies of the structure–activity relationship at different pH values and temperatures revealed its high functional and structural stability. The inhibitory activity demonstrated on cysteine proteases bromelain, ficin and cathepsin L, and the cysteine proteases of the larval midguts of *C. maculatus* suggests other endogenous roles for 2S albumin. AaCI-2S inhibited cell proliferation of gastric cancer and two lines of prostate cancer and did not affect the proliferation of non-tumorigenic cells. As the seeds of *Araucaria* are used as food, its antiproliferative effect is of nutritional significance for future studies and offers evidence regarding the benefits of including the pine nut as a functional food in our diet.
